# Lipidome of extracellular vesicles from *Giardia lamblia*

**DOI:** 10.1371/journal.pone.0291292

**Published:** 2023-09-08

**Authors:** Clarissa Perez Faria, Barbara Ferreira, Ágata Lourenço, Inês Guerra, Tânia Melo, Pedro Domingues, Maria do Rosário Marques Domingues, Maria Teresa Cruz, Maria do Céu Sousa

**Affiliations:** 1 Center for Neuroscience and Cell Biology, University of Coimbra, Coimbra, Portugal; 2 Faculty of Pharmacy, University of Coimbra, Coimbra, Portugal; 3 Department of Chemistry, CICECO Aveiro Institute of Materials, University of Aveiro, Aveiro, Portugal; 4 Department of Chemistry, CESAM Centre for Environmental and Marine Studies, University of Aveiro, Aveiro, Portugal; 5 Mass Spectrometry Centre, LAQV-REQUIMTE, Department of Chemistry, University of Aveiro, Aveiro, Portugal; I3S: Universidade do Porto Instituto de Investigacao e Inovacao em Saude, PORTUGAL

## Abstract

Extracellular vesicles (EVs) (exossomes, microvesicles and apoptotic bodies) have been well acknowledged as mediators of intercellular communications in prokaryotes and eukaryotes. Lipids are essential molecular components of EVs but at the moment the knowledge about the lipid composition and the function of lipids in EVs is limited and as for now none lipidomic studies in *Giardia* EVs was described. Therefore, the focus of the current study was to conduct, for the first time, the characterization of the polar lipidome, namely phospholipid and sphingolipid profiles of *G*. *lamblia* trophozoites, microvesicles (MVs) and exosomes, using C18—Liquid Chromatography—Mass Spectrometry (C18-LC-MS) and Tandem Mass Spectrometry (MS/MS). A total of 162 lipid species were identified and semi-quantified, in the trophozoites, or in the MVs and exosomes belonging to 8 lipid classes, including the phospholipid classes phosphatidylcholine (PC), phosphatidylethanolamine (PE), phosphatidylglycerol (PG), phosphatidylinositol (PI), cardiolipins (CL), the sphingolipid classes sphingomyelin (SM) and ceramides (Cer), and cholesterol (ST), and 3 lipid subclasses that include lyso PC (LPC), lyso PE (LPE) and lyso PG (LPG), but showing different abundances. This work also identified, for the first time, in *G*. *lamblia* trophozoites, the lipid classes CL, Cer and ST and subclasses of LPC, LPE and LPG. Univariate and multivariate analysis showed clear discrimination of lipid profiles between trophozoite, exosomes and MVs. The principal component analysis (PCA) plot of the lipidomics dataset showed clear discrimination between the three groups. Future studies focused on the composition and functional properties of *Giardia* EVs may prove crucial to understand the role of lipids in host-parasite communication, and to identify new targets that could be exploited to develop novel classes of drugs to treat giardiasis.

## Introduction

*Giardia lamblia* (syn. *Giardia intestinalis*, *Giardia duodenalis*) is one of the most common causes of parasitic gastrointestinal infection worldwide, with an estimated 280 million cases of symptomatic giardiasis annually [1]. The parasite has two development forms: trophozoites, which colonizes the intestine, and the cyst form, which is excreted in feces and spreads the parasite. Cysts in the environment, especially in water, are very resistant and can cause diarrhea outbreaks when ingested [2]. The spectrum of clinical manifestations of giardiasis is quite variable, ranging from asymptomatic infections to acute or chronic diarrhea [3, 4]. The most common clinical signs of infection are diarrhea (with or without malabsorption syndrome), bloating, vomiting, nausea, dehydration, abdominal pain, flatulence, and weight loss [5]. *Giardia* trophozoites attach strongly to the intestinal epithelial cells via a ventral adhesive disc and cause significant damage and disruption to gastro epithelial cells in the absence of cell invasion and secreted toxins [6].

Several studies pointed out that *Giardia* shedding extracellular vesicles (EVs), which participate in cellular communication between *Giardia* and the host cells, could modulate the physiopathology of giardiasis and immune response [7–12]. These studies suggest that the interaction between *Giardia* EVs and host cells at multiple molecular levels is crucial to established the infection (in review by [13].

EVs are nanovesicles enclosed by a lipid bilayer that are released by prokaryotic and eukaryotic cells, both healthy and diseased. They containing *in cargo* a variety of compounds, including proteins, DNA, RNA, non-coding RNA, lipids and metabolites [14]. In 2012, the first International Society of Extracellular Vesicles grouped EVs into three categories based on their releasing pathways, size and how they sediment. Apoptotic bodies are a heterogeneous group of particles that are larger than 2 μm and pelleted at 2000–10,000 x g. Microvesicles range from 100 nm to 1 μm and pelleted at 10,000–20,000 x g. Exosomes are the smallest particles, measuring less than 100 nm and pelleted at > 10,000 x g (reviewed in [15].

The content of each EVs is determined by its biogenesis and the type of cell from which it is secreted. Furthermore, the content can be affected by the physiological or pathological condition of the cell [16, 17]. Exosomes are released from late endosomal microvesicular bodies (MVBs), upon the fusion of MVBs with a cell’s plasma membrane [18, 19], while microvesicles (also called ectosomes) are originate from cell surface by diret plasma membrane budding (reviewed in [15].

Lipids are essential molecular components of EVs, with regulatory and structural functions, being essential components during their biogenesis and release into the extracellular environment [20]. Studies performed on mammalian EVs have demonstrated that lipid species are often enriched compared to the cells of origin, with increased levels in cholesterol, sphingolipids, glycosphingolipids, and phosphatidylserine [21].

It is well documented that *Giardia* has a limited ability to synthesize *de novo* its own lipid molecules and, therefore, depends on exogenous lipids for its growth and differentiation [22, 23]. Therefore, many of these exogenous lipids undergo remodeling and/or base-exchange reactions before they are incorporated into giardial membranes [24]. Indeed, *Giardia* has evolved mechanisms to import exogenous lipids and cholesterol by receptor-mediated endocytosis and traffic via clathrin-mediated and actin/microtubule-dependent pathways [25]. The predominant sphingolipids (SLs) in *Giardia* are sphingoid bases (sphingosine and sphinganine), ceramides, GSLs and sphingomyelin SM [22]. The levels of SLs are differentially regulated during encystation and ceramide-1-phosphate (Cer-1-P) is a newly generated lipid in *Giardia* [22]. It seems that the Cer-1-P is generated from sphingosine, possibly by remodeling reactions and subsequently is released. A recent lipidomic analysis has show that the lipid and fatty acid profiles of Giardia undergo dynamic changes throughout its life stages [26, 27]. In the cyst form of *Giardia* is possible to find several lipid classes including, LPC, P- and O-LPC, LPE, DG, TG, CE, PC, P-PC (Plasmalogen), and ceramides. In contrast, lipids such as SM, PG, BMP were found predominantly in encysting trophozoites. Additionally, ether linked PC species were found to be the most abundant in trophozoites.

At the moment the knowledge about the lipid composition and the function of lipids in EVs is limited [28, 29] and as for now none lipidomic studies in *Giardi*a EVs was described. Therefore, and going behind the state of the art, this study aims to profile the lipid components of the *G*. *lamblia* trophozoites and of two populations of *G*. *lamblia* EVs, exosomes and microvesicles, using Mass Spectrometry—based lipidomics (MS-based lipidomics).

## Material and methods

### Chemicals

Organic solvents (dicloromethane, methanol, acetonitrile and isopropanol), purchased from Fisher Scientific (Leicestershire, UK), were HPLC grade and used without further purification Perchloric acid (HClO_7_, 70%) from Chem-Lab NV (Zedelgem, Belgium) and ammonium molibdate tetrahydrate ((NH_4_)_6_Mo_7_O_24_·4H_2_O) from Panreac (Barcelona, Spain), ascorbic acid (C_6_H_8_O_6_) from VWR International (Leicestershire, UK), sodium dihydrogen phosphate dihydrate (NaH_2_PO4·2H_2_O) from Riedel-de Haёn (Seelze, Germany). MilliQ water was used, filtered through a 0.22 mm filter in a Milli-Q Millipore system (MilliQ plus 185). Phospholipids and ceramide internal standards used were (1,3-bis[1,2-dimyristoyl-sn-glycero-3-phospho]-sn-glycerol) (CL(14:0)_4_), 1,2-dimyristoyl-sn-glycero-3-phosphocholine (dMPC), 1-nonadecanoyl-2-hydroxy-sn-glycero-3-phosphocholine (LPC(19:0)), 1,2-dimyristoyl-sn-glycero-3-phosphoethanolamine (dMPE), 1,2-dimyristoyl-sn-glycero-3-phosphate (dMPA), 1,2-dimyristoyl-sn-glycero-3-phospho-(10-rac-glycerol) (dMPG), 1,2-dimyristoyl-sn-glycero-3-phospho-L-serine (dMPS), 1,2-dipalmitoyl-sn-glycero-3-phospho-(10-myo-inositol) (dPPI), N-heptadecanoyl-D-erythro-sphingosylphosphorylcholine (SM(17:0/d18:1)) and N-heptadecanoyl-D-erythro-sphingosine (Cer(17:0/d18:1)), purchased to Avanti polar lipids Inc (Alabaster, AL).

### Production and characterization of *Giardia* extracellular vesicles (EVs)

*Giardia lamblia* trophozoites (strain WB, clone 6 [ATCC 30957] were maintained in axenic culture at 37°C in 10 ml of Keister’s modified TYI-S-33 medium, in microaerophilic conditions and subcultured when confluent [30, 31]. *Giardia* EVs production was stimulated by CaCl_2_ inducer (1 mM) in free-exossomes culture medium (without serum) and the isolation of EVs was achieved by differential ultracentrifugation [7, 8]. Briefly, *G*. *lamblia* cells in log-phase of growth were washed twice with warm PBS 1X (37°C) to eliminate detached and dead parasites. Adherent parasites were collected by cooling of the culture vials on ice for 20 min and centrifuged at 400 x g for 5 min at 4°C. After that, *G*. *lamblia* was counted in a Neubauer cell-counter chamber and diluted to 1 × 10^6^ parasites/mL in TYI-S-33 medium without bovine serum in order to avoid serum-derived exosome contamination. 1mM of CaCl_2_ was added for EVs induction. The parasites were incubated at 37°C for 1 h for EVs releasing. Then, the culture supernatants were subjected to successive centrifugation steps at 320 × g (10 min) to remove the cells, and then for 4,000 × g/30 min to remove cellular debris. Next, the supernatants were centrifuged at 15,000 × g (1 h) and the resulting microvesicles-containing pellet was resuspended in phosphate buffered saline (PBS). The remaining supernatant was then ultracentrifuged for 100,000 × g for 4 h, and the resulting Exossomes-containing pellets were resuspended in PBS (1x). Both samples were filtrated by membrane filter (0.45 μm) and kept at -80°C. EVs samples were firstly quantified based on their protein concentrations using the microBCA assay (ThermoFisher™). Quantification and size distribution of isolated EVs was performed by Nanosight tracking analysis, using a NanoSight LM 10 instrument (NanoSight Ltd), and transmission electron microscopy was performed for ultrastructural characterization of *Giardia* EVs.

### Extraction of lipids

The cell and EVs pellets were resuspended in 1mL of ultra-pure water (Milli-QH_2_O). The pellets were washed twice with ice-cold Milli-QH_2_O, and centrifuged. Thereafter, the total lipids were extracted according to the method of Bligh and Dyer [32]. Briefly, 3.75 mL of a mixture of dichloromethane/methanol 1:2 (v/v) was added to the cell and EVs homogenate, vortexed and incubated on ice for 30 minutes. An additional 1.25 mL of chloroform was added followed by vortexing. Then 1.25 mL Milli-Q H_2_O were added, and vortexed. The samples were centrifuged at 1000 rpm for 5 min at room temperature to obtain a two-phase system: an aqueous upper phase and an organic lower phase. The lipid extract was recovered from the organic phase. To ensure complete extraction of the lipid phase, 1.88 mL of chloroform were added to the aqueous phase, followed by vortexing and re-centrifugation. The organic phases were recovered in a glass vial and dried with a stream of nitrogen. After drying, the total lipid extracts were re-suspended in 300 μL of chloroform, transferred to a vial, dried under a stream of nitrogen and stored at -80°C.

### Quantification of phospholipids content by phosphorus assay

To determine the total amount of phospholipids (PLs) in each lipid extract, phosphorus determination was performed according to Bartlett and Lewis [33]. Detailed experimental procedures have been previously described by Guerra *et al*. [34]. The extracts enriched in PL were dissolved in 200 μL of dichloromethane, and a volume of 10 μL was transferred, in duplicate, to a glass tube, previously washed with 5% nitric acid. The solvent was dried under a stream of nitrogen, and a volume of 125 μL of 70% perchloric acid was added to each tube. Samples were incubated in a heating block (Stuart, U.K.) for 1 hour at 180°C. After cooling to room temperature, a volume of 825 μL of Milli-Q water, 125 μL of ammonium molybdate (25 g L-1 prepared in Milli-Q water), and 125 μL of ascorbic acid (100 g L-1 prepared in Milli-Q water) were added to each sample, with vortex mixing between each addition. The samples were then incubated in a water bath at 100°C for 10 min. Then the samples were immediately cooled in a cold-water bath. The absorbance was measured at 797 nm in a Multiskan GO1.00.38 Microplate Spectrophotometer (Thermo Scientific, Hudson, NH, USA) controlled by SkanIT software, version 3.2 (Thermo Scientific). The P content of each extract was determined from a standard curve prepared by performing the same procedure (without the heat block step) with standards containing 0.1 to 2 μg of P prepared from a solution of sodium dihydrogen phosphate dihydrate (100 μg mL-1 of P). The total amount of PL was then estimated by multiplying the amount of P by 25 [35].

### C18-LC-MS and MS/MS

Lipid extracts were analysed by reverse-phase liquid chromatography in a Dionex Ultimate 3000 (Thermo Fisher Scientific, Bremen, Germany) using an Ascentis® Express C18 column (Sigma-Aldrich®, 2.1 x 100 mm, 2.7 μm) coupled to the Q-Exactive® hybrid quadrupole Orbitrap mass spectrometer (Thermo Fisher, Scientific, Bremen, Germany). Mobile phase A was water/acetonitrile (40/60%) with 10 mM ammonium formate and 0.1% formic acid. Mobile phase B was isopropanol/acetonitrile (90/10%) with 10 mM ammonium formate and 0.1% formic acid. The following gradient was applied: 32% B at 0 min, 45% B at 1.5 min, 52% B at 4 min, 58% B at 5 min, 66% B at 8 min, 70% B at 11 min, 85% B at 14 min, 97% B at 18 min, 97% B at 25 min, 32% B at 25.01 min and 32% B at 33 min. A volume of 5 μL of each sample, containing 10 μg of lipid extract (in 10 μL of dichloromethane), 82 μL of a solvent system composed of 50% isopropanol/ 50% methanol, and 8 μL of a mixture of phospholipid standards (dMPC—0.04 μg, SM d18:1/17:0–0.04 μg, dMPE—0.04 μg, LPC—0.04 μg, dPPI—0.08 μg, CL(14:0)4–0.16 μg; dMPG—0.024 μg, Cer(17:0/d18:1) - 0.08 μg, dMPS—0.08 μg; dMPA-0.16 μg), was loaded into the column at 55°C and at a flow-rate of 260 μL min−1. The mass spectrometer operated in simultaneous positive (ESI 3.0 kV) and negative (ESI −2.7 kV) modes as previously described. The capillary temperature was 320°C and the sheath gas flow was 35 U. Data was acquired in full scan mode with a high resolution of 70,000, automatic gain control (AGC) target of 3 x 106, in an m/z range of 300–1600, 2 microscans, and maximum injection time (IT) of 100 ms. Tandem mass spectra (MS/MS) were obtained with a resolution of 17,500, AGC target of 1x105, 1 microscan, maximum IT of 100 ms. The cycles consisted of a full-scan mass spectrum and ten data-dependent MS/MS scans, which were repeated continuously throughout the experiments with a dynamic exclusion of 30 s and an intensity threshold of 8 x 104. The normalized collision energy (CE) ranged between 20, 24 and 28 eV in the negative mode and 25 and 30 eV in the positive mode. Data acquisition was performed using the Xcalibur data system (V3.3, Thermo Fisher Scientific, Bremen, Germany).

Lipid species were identified using mass spectrometry-data independent analysis (MSDIAL) v4.70 software [36]. Identification of the lipid species was performed in the negative and positive ionisation modes, using the raw files acquired in MS/MS mode, and converted by the ABF converter (https://www.reifycs.com/AbfConverter/ (accessed on 13 February 2022)) compared to the lipid database provided by the MS-DIAL software. The tolerances for MS and MS/MS search were set at 0.01 Da and 0.05 Da, and all identifications were verified manually. The validated species were integrated and quantified in the Mzmine v2.53 software [37]. Raw LC-MS files were subjected to smoothing and filtering methods, peak detection (including chromatogram construction, peak deconvolution and deisotoping), and peak alignment with gap filling. Later, the integrated peak areas of the extracted ion chromatograms (XIC) were exported, and the normalisation was obtained by calculating the ratio against a selected internal lipid standard with from the same lipid class and subclass. The normalization of the two subclasses LPE and LPG was made using the internal standard LPC(19:0), and for the ST class was using the dMPC, since they had a nearest retention time.

### Data analysis and statistics

Multivariate and univariate analyses were performed using R version 3.5 in Rstudio version 1.1.4. After imputation of missing values with a small value, data was log-transformed and normalized with EigenMS [38] using R v4.1.0. Principal Component Analysis (PCA) was performed for the exploratory data analysis, with the R package FactoMine [39]. After verifying normality by visual inspection of the quantile-quantile plots against the normal distribution, a one-way ANOVA (ANalysis Of VAriance) analysis with post-hoc Tukey HSD (Honestly Significant Difference), was performed with the R built-in function. Heatmaps were created after selecting the 50 most significantly different PLs using the R package pheatmap [40] using "Euclidean" as the clustering distance, and "ward.D" as the clustering method. All plots were created using the R package ggplot2 [41] and ggpubr [42].

## Results

### Characterization of *Giardia* EVs

**Fig 1:**
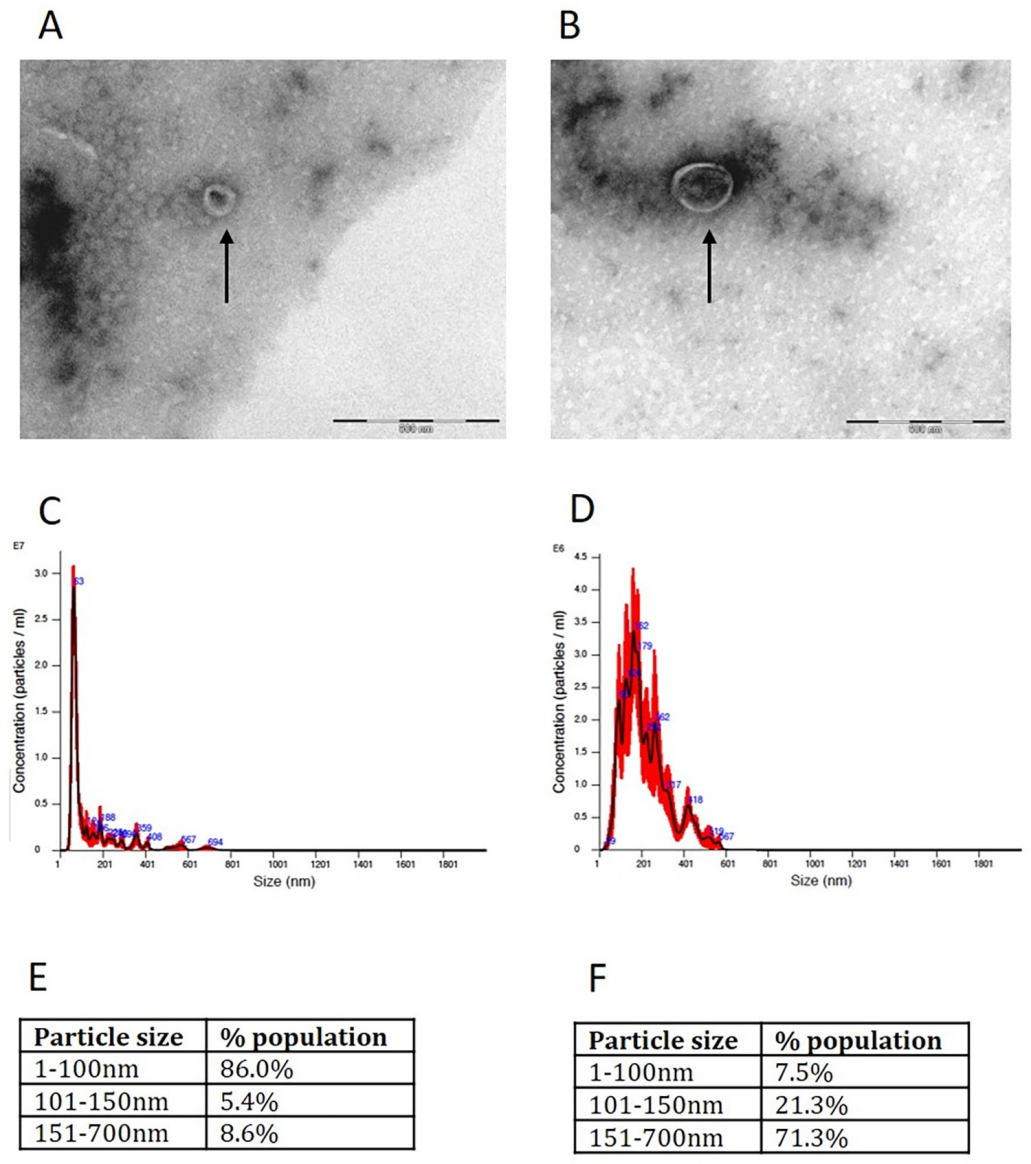
Characterization of *Giardia lamblia* extracellular vesicles (EVs) by Transmission Electron Microscopy (TEM) (A, B) and Nanoparticle tracking analysis (NTA, Nanosight) (C-F). Trophozoites of *Giardia lamblia* (ATCC 50803 WB clone C6) were culture in TYI medium without serum and supplemented with CaCl_2_ (1 mM) to produce *Giardia* EVs. The EVs were isolated by sequential ultracentrifugation. For TEM analyses, the samples were fixed in 1% gluteraldehyde and deposited onto carbon-coated grids. For the quantification of EVs diameter by NanoSight, each sample was diluted 1:100 in PBS (1x) and subjected to a NS300 Nanosight with readings performed in triplicate during 60 s videos at 10 frames per second at room temperature, with the following parameters: camera shutter −1492, camera gain −512, detection threshold −10. Size analysis of EVs by TEM shows that the 100.000 x g fraction contains particle sizes smaller than 100nm (exosomes) (A) and that the 15.000 x g fraction contains particles larger than 100nm (MVs) (B). Scale bar: 500nm. C, D) Estimated size distribution profiles of exosomes (C) and MVs (D) using nanoparticle tracking system (Nanosight). (E, F) Tables from Nanosight analysis of percentage of purified vesicles in various size ranges for exosomes (E) and MVs (F).

The *Giardia* EVs were characterized by transmission electron microscopy (TEM) and Nanoparticle tracking analysis (NTA, Nanosight) (Fig 1). Analysis of EVs using TEM revealed cup-shaped vesicles of 50–90 nm (exosomes) (Fig 1A) and 117–282 nm (MVs) (Fig 1B). The nanosight analysis showed that the size of exosomes peak with a mean diameter of 82.6nm, 86% are <100 nm in size and concentration was 1,11 × 10^11^ particles/mL (Fig 1C and 1E). Whereas the size of MVs peak with an average diameter of 238.5 nm, 92.5% are between 100–700 nm in size and concentration was 1,9 × 10^10^ particles/mL (Fig 1D and 1F). We also stained *Giardia* EVs populations with PKH26, a lipophilic membrane dye, and characterized the of EVs proteins by proteomic analysis showing the presence of the lipid-bilayer structure specific of EVs and of *Giardia* specific proteins (Elongation factor 1-alpha, Alpha-7.3 giardin, tubulin and Variant Surface Proteins (VSPs) (patent PCT/IB2022/057466; ref. P1303-7WO).

### Lipidome of *Giardia* trophozoites and EVs

**Fig 2:**
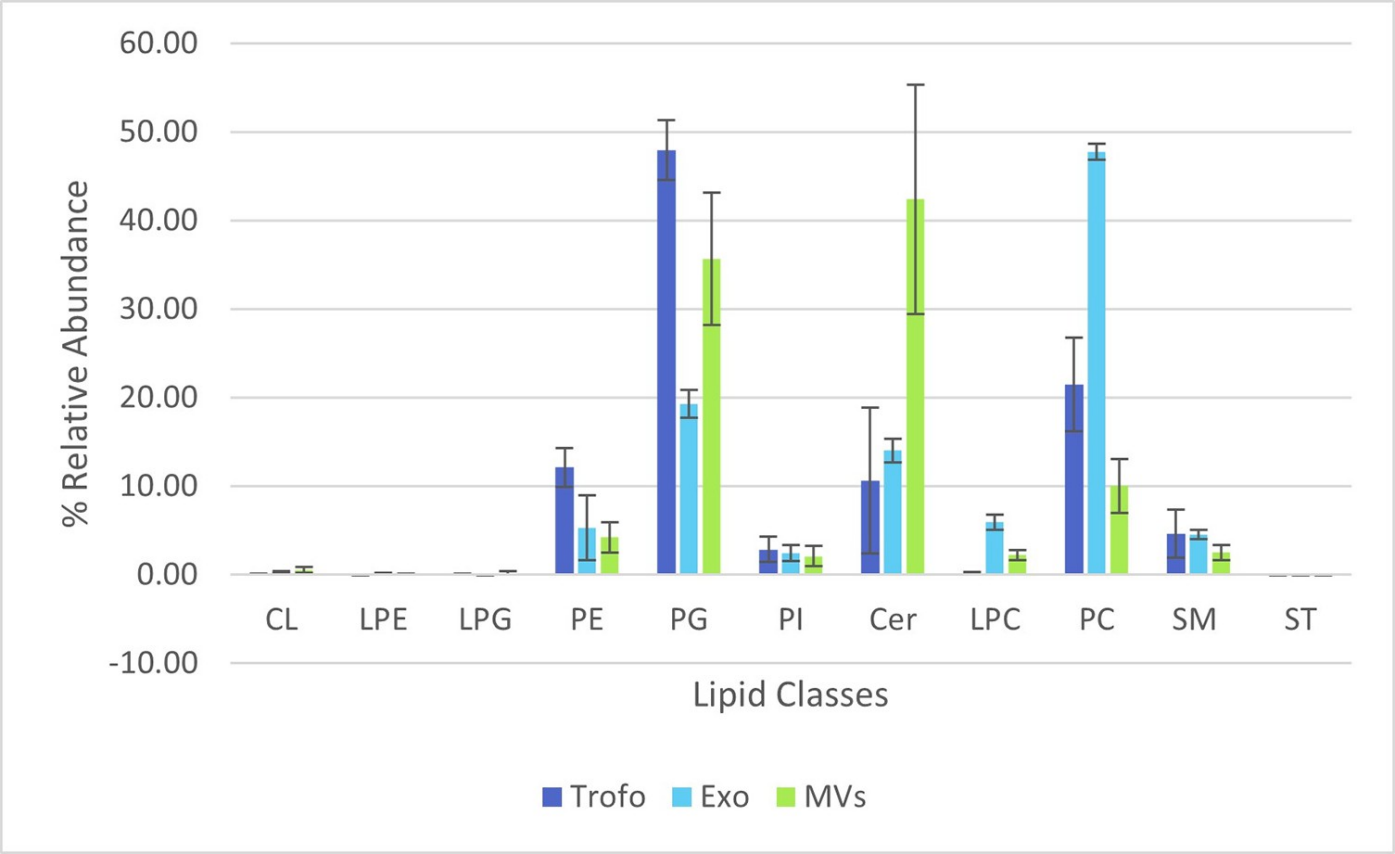
Representation of the relative abundance (%) of each class of lipids (calculated by dividing each lipid class (μg) (calculated by the sum of all lipid species per class) per sum of all lipid classes (μg).

The lipidome of the total lipid extracts obtained from *G*. *lamblia* trophozoites (Tropho), microvesicles (MVs) and exosomes (Exo), was analysed by C18-LC-MS and MS/MS high resolution mass spectrometry. A total of 162 lipid species (*m/z* values) were identified and semi-quantified, belonging to 8 lipid classes, including the phospholipid classes phosphatidylcholine (PC), phosphatidylethanolamine (PE), phosphatidylglycerol (PG), phosphatidylinositol (PI), cardiolipins (CL), the sphingolipid classes sphingomyelin (SM) and ceramides (Cer) and cholesterol (ST), and lipid subclasses such as lyso PC (LPC), lyso PE (LPE) and lyso PG (LPG) (S1 Table). The lipidome comprises 49 PCs (including diacyl, alkyl/alkenyl-acyl species), 10 LPCs (including monoacyl, alkyl/alkenyl species), 19 PEs (including diacyl, alkyl/ alkenyl-acyl species), 2 LPEs, 16 PGs, 1 LPG, 5 PIs, 3 CLs, 19 SMs, 37 Cer and 1 ST. All lipid species were identified in the three groups, but with different relative abundances in each class as is howed in the profile per classes (Fig 2). Details of representative MS/MS spectra of lipids species and of the relative abundance (%) of each class of lipids could be consulted in supplementary informations (S1–S14 Figs). The PG class was the most abundant in Tropho, while PG and Cer were the most abundant in MVs and PC the most abundant phospholipod class in Exo. The lipid species were esterified to fatty acyl chains with a carbon number from 14 to 26 and with double bonds between 0 and 6.

### Comparation of lipid profiles in trophozoites, exosomes and MVs

**Fig 3:**
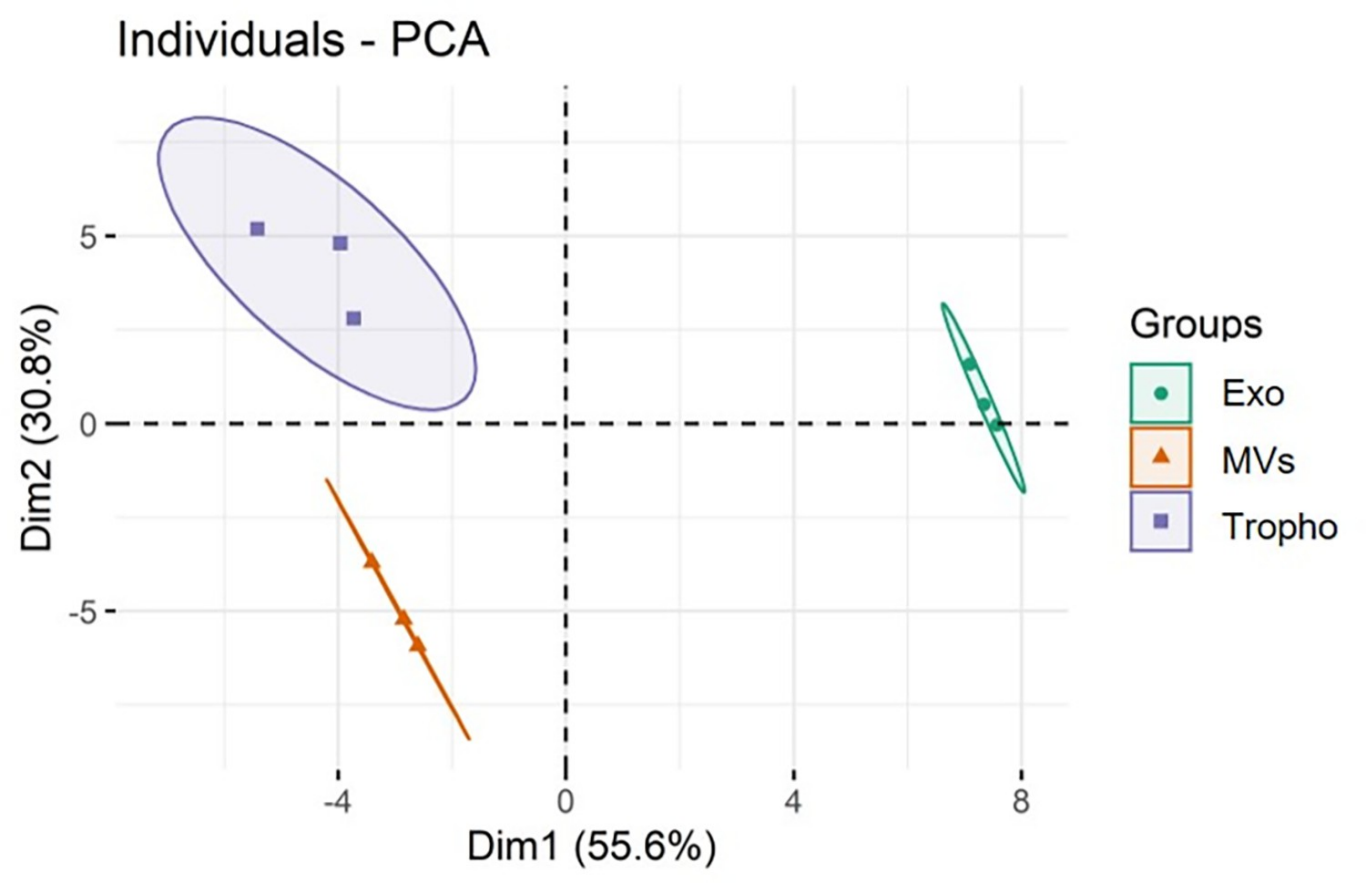
PCA score plot of the phospholipid and sphingolipids species dataset obtained by C18-LC−MS/MS analysis of total lipid extracts obtained from *Giardia lamblia* trophozoites (Tropho), microvesicles (MVs) and exosomes (Exo).

Lipid profiles were then compared between the three groups (Tropho, Exo and MVs) using univariate and multivariate analysis. The principal component analysis (PCA) plot of the lipidomics dataset showed clear discrimination between the three groups (Fig 3). The eigenvalues of the first two principal components accounted for 86.4% of the total variance (Dim1 55.6%; Dim2 30.8%). The Exo group was clustered in the right region of the plot, while the Tropho and MVs groups were more associated, but discriminated along the Dim2.

**Fig 4:**
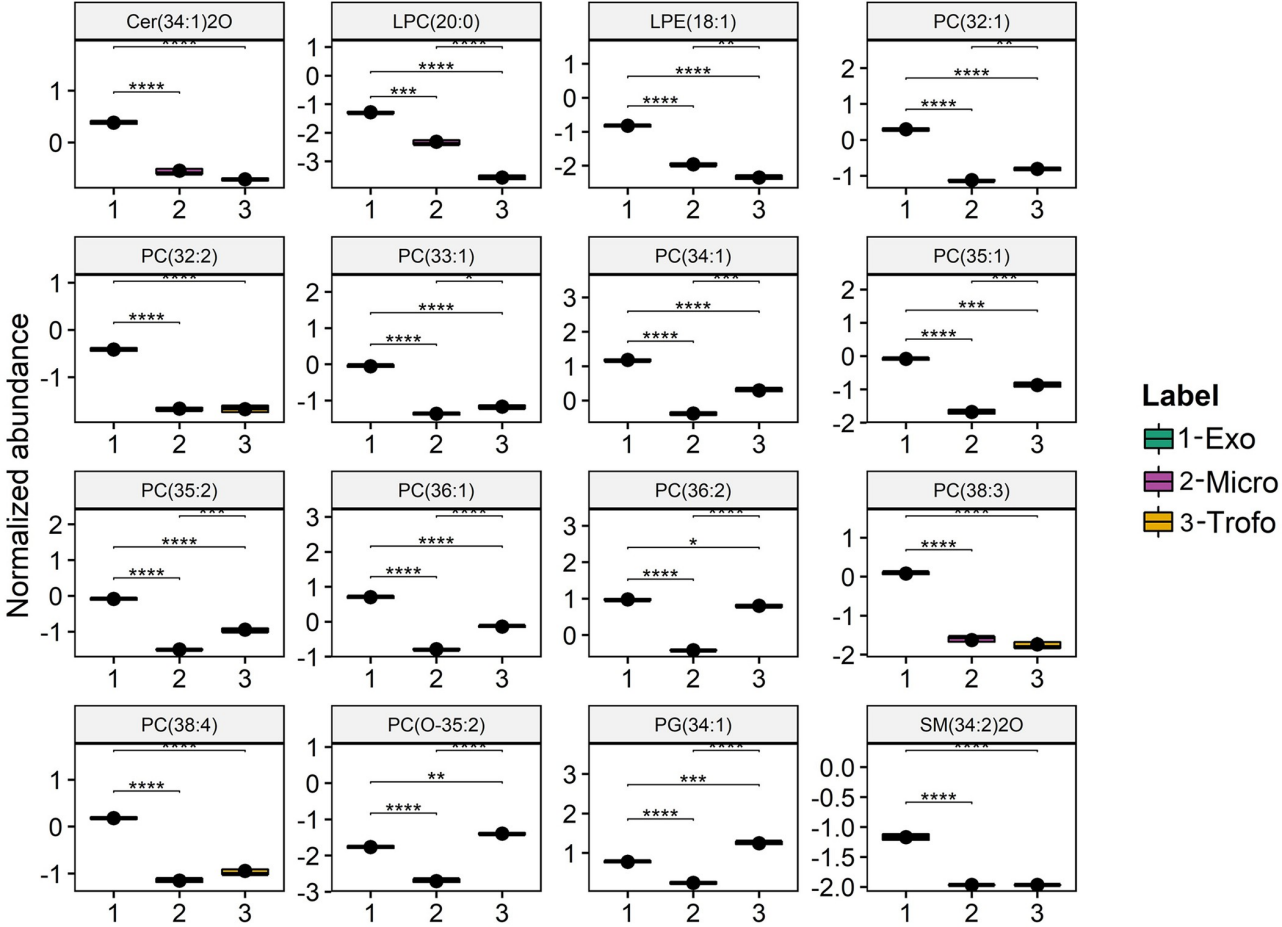
Boxplots of the 16 main lipid species showing the major variation between exosomes (Exo) (1), microvesicles (MVs (2)) and *Giardia lamblia* trophozoites (Tropho) (3 = groups sorted (left to right, top to bottom) by the lower *p*-values of ANOVA. The lipid species displayed in the figure are annotated with the *q*-value from the Tukey’s post hoc tests:****<0.0001, *** < 0.001, **<0.01 and *<0.05. The phospholipid species are labelled as follows: AAA(xx:i) (AAA = lipid class abbreviation; xx = number of carbon atoms in fatty acid(s); i = number of double bonds). Lipid classes abbreviations: Cer, Ceramide; LPC, lysophosphatidylcholine; LPE, Lysophosphatidylethanolamine; PC, phosphatidylcholine; PG, phosphatidylglycerol; SM, sphingomyelin.

Univariate analysis (ANOVA followed by Tukey’s post-hoc test) was also performed on the transformed dataset of the different species of phospholipids, sphingolipids and sterols, to test the association of these variables with the three clusters (Tropho, MVs and Exo). This analysis showed that 123 of the 162 lipid species were significantly different with *p*<0.05, between the three groups. Boxplots of the 16 major species with the lowest *p*-values (*p*-value < 0.05) are shown in Fig 4. The distribution of the 16 lipid species that showed major variation is as follows: 10 PC, 1 PC-O, 1 LPC, 1 LPE, 1 PG, 1 SM and 1 Cer. All species were significantly increased in Exo, except PG 24:1 and PC O-35:2 which were significantly increased in the MVs group, contributing to the differentiation between these two groups.

**Fig 5:**
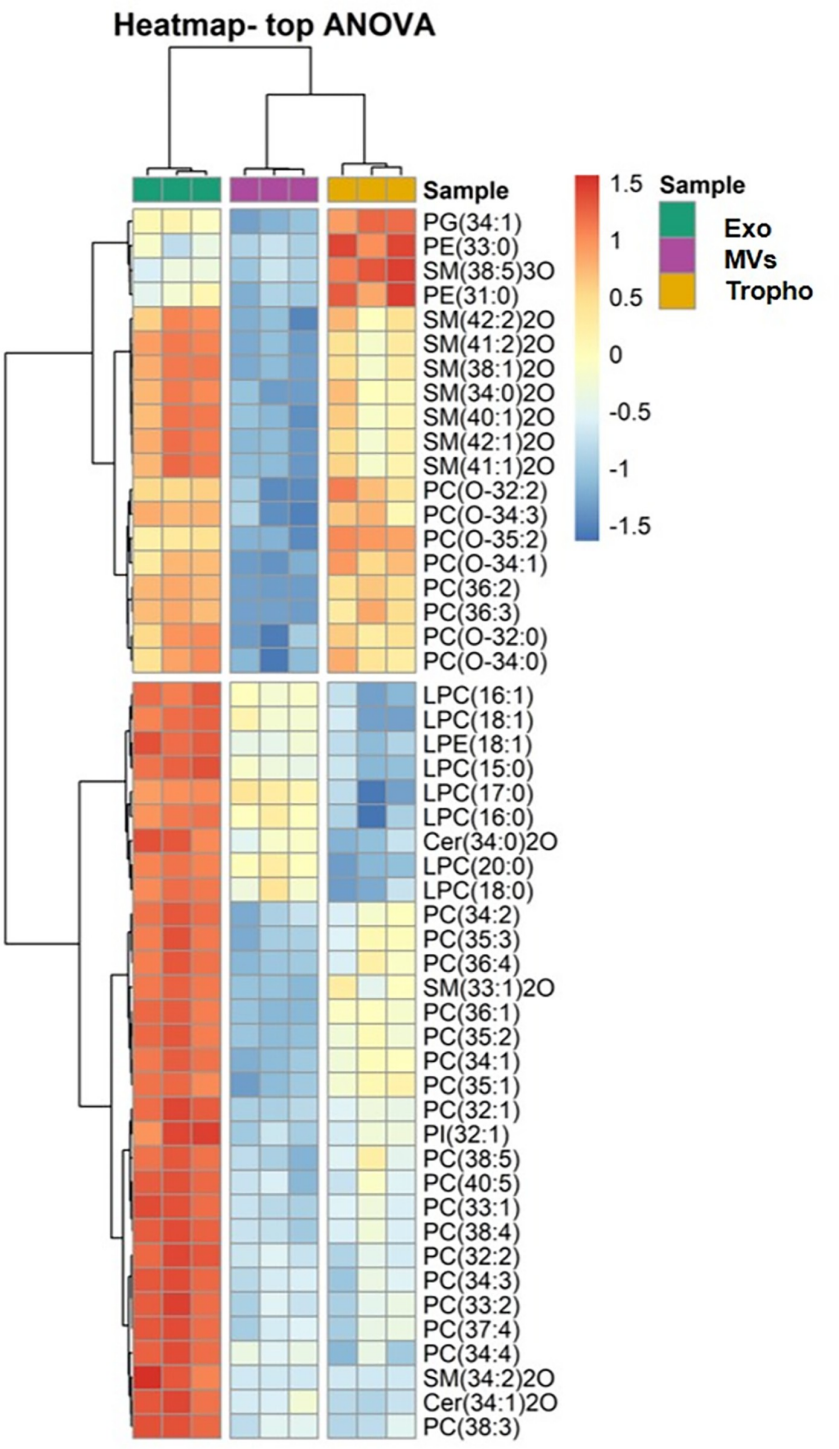
Two-dimensional hierarchical clustering heat map of the 50 main lipids, of the exosomes (Exo), microvesicles (MVs) and *Giardia lamblia* trophozoites (Tropho) groups. The dendrogram on the top shows the clustering of samples, and on the left shows the clustering of lipid species. Levels of relative abundance are shown on the colour scale, with numbers indicating the fold difference from the mean. The lipid species are indicated as follows: AAA (xx:i) (AAA = lipid class; xx = number of carbon atoms in FA(s); i = number of double bonds). Lipid classes abbreviations: PG, phosphatidylglycerol; PE, phosphatidylethanolamine; SM, sphingomyelin; PC, phosphatidylcholine; LPC, lysophosphatidylcholine; LPE, lysophosphatidylethanolamine; Cer, ceramide; PI, phosphatidylinositol.

We used the information from the univariate analysis to create a heatmap dendrogram with two-dimensional hierarchical clustering, using the first 50 lower *p*-values (*p-*values < 0.05) from the ANOVA test (Fig 5).

Fig 5 showed, in the primary split of the upper hierarchical dendrogram, that the samples clustered independently in the three groups. The separation of the three groups was consistent with what was shown in the PCA score plot (Fig 3). The clustering of individual lipid species for their similarity in changes in lipid expression groups clearly showed two groups (left of the heatmap in Fig 5). From top to bottom, the first level of the dendrogram included 19 lipid species (8 SM, 6 PC-O, 2 PC, 2 PE, 1 PG), and these species had a higher content in Exo and Tropho samples, compared with MVs samples. But there are 4 lipid species (PG 34:1, PE 33:0, SM 38:5;2O and PE 31:0) that showed higher content in Tropho samples compared to Exo, while the other 7 SM species were more abundant in Exo, allowing for discrimination between the two groups. The second group contained a total of 31 lipid species, namely 18 PC, 7 LPC, 1 LPE, 1 PI, 2 Cer and 2 SM, all more abundant in the Exo group. Between the Tropho and MVs groups, lysophospholipids (7 LPC and 1 LPE) and Cer 34:0;2O were higher more abundant in the MVs samples, although with lower intensity compared to Tropho samples.

## Discussion

Over the last few years, studies of EVs, including exosomes and microvesicles (MVs), have attracted considerable interest in the scientific community due to their prominent function in intercellular communication and involvement in a wide range of physiological and pathological roles [18, 43]. EVs could be explored for the diagnostic, as potential biomarkers of different diseases, and could act as drug-delivery carriers. EVs are also promising as a therapeutic tool due to their natural capacity to carry bioactive molecules such as proteins, lipids and nucleic acids [43, 44].

While many studies have demonstrated the EVs potential for different purposes, it was only recently that the characterization of *G*. *lamblia* EVs and their role in the host-parasite interaction began to be studied [7, 8, 11]. Evans-Osses and collaborators demonstrated that *G*. *lamblia* trophozoites are able to release MVs in response to different pH levels and calcium (vesicle inducer) [7]. Subsequent study of Gavinho and collaborators described two distinct EV populations from *G*. *lamblia* where large EVs are associated with effective parasite adhesion to the host [8]. It was also suggest that one of the mechanisms by which *G*. *lamblia* secretes and transports proteins involves extracellular vesicles (EVs) [45]. Zhao and colleagues showed that *Giardia* EVs could modulate the host cell innate immunity [11]. Our team work also demonstrated that Giardia EVs evoke a pro-inflammatory profile on macrophages cells and DCs and induce adaptive immune response in mice (patent PCT/IB2022/057466; ref. P1303-7WO). Also, a bacteriostatic effect was observed when exposed *Giardia* EVs with commensal gut bacteria [46].

Although the proteome of *Giardia* EVs had been characterized so far [7, 8] their lipidomic profile remains unexplored, and constitutes the main goal of this paper. Therefore, using lipidomics based on C18-LC-MS and MS/MS high-resolution mass spectrometry, we were able to identify, for the first time, the polar lipidome, namely the phospholipid and sphingolipid profiles of *G*. *lamblia* trophozoites (Tropho), microvesicles (MVs) and exosomes (Exo).

A total of 162 lipid species belonging to 8 phospholipid classes, 2 sphingolipid classes and 1 sterol class were identified either in the tropho group, or in the MVs and Exo groups. The same classes of lipids were identified in the three group samples, nevertheless in distinct proportions. The PG class was quite abundant in Tropho and MVs, being the most abundant in Tropho, and PC the most abundant class in Exo. Cer class was quite abundant in MVs than in other extracellular vesicles. Interestingly, some publication described MVs to be enriched in this lipid class, while Exo to be enriched in glycolipids and free fatty acids [47]. However, the few papers in this area do not allow us to take many conclusions.

On the other hand, published works reports on membrane lipids from microvesicles [48]. Perhaps, these microvesicles can be carriers of ceramides and thus the higher abundance of this lipid class can be understood. In addition, the lower amount of SM found in the MVs samples, may be associated to its degradation in Cer by the action of sphingomyelinases [49, 50]. These abundant lipids classes in the MVs samples, like PG and Cer can provide a potential pool of lipids that can be scavenged by the parasite and possibly be used to interact with host cells and other microorganisms of the gut microbiota, although more studies should be performed focused on the functional role of these lipids in an infection context.

This work also identified, for the first time, the lipid classes of LPC, LPE, LPG, CL, Cer and ST in *G*. *lamblia* trophozoites. Indeed, the *G*. *lamblia* lipidome has already been described, but the identification was made several years ago without using the new high resolution-based lipidomics approaches currently available, leaving out many important lipid species to identify [22, 23, 51–53]. Previous studies have identified the lipid classes of PC, SM, PE, PG, and PI as the major lipid classes of the parasite *G*. *lamblia* [23, 51–53].

Concerning the lipidome and profiling at molecular level of MVs and exosomes from *G*. *lamblia*, it has never been disclosed so far. However, the lipidome of these two extracellular vesicles has already been identified for other parasites and different cell sources [47, 54–57] Microvesicles are known to originate from the extrusion of the plasmatic membrane of *G*. *lamblia* with the bidding of the small plasma membrane domain. In contrast, exosomes arise from the peripheral vacuoles (PVs), the components of the endo-lysosomal system in *G*. *lamblia*, with a participation of the ER [58]. Using high-resolution electron microscopy, it was found that intraluminal vesicles (ILVs) are present inside some of the PVs, suggesting that those organelles could also act as multivesicular bodies (MVBs) [59]. The exosomes production and release is dependent on ESCRT-associated protein Vps4a, Rab 11, and ceramide. Rab 11 protein plays a role in cell differentiation and division, and is also involved in the communication between the ER and the VPs. Furthermore, the exocytic vesicular trafficking related Rab 1 and Rab 2 a/b proteins, are found to be implicated in the exosome exocytosis process (reviewed in [13]). Therefore, it is expectable that the same lipid species in the MVs group was similar to that of *G*. *lamblia* trophozoites compared to the Exo group. However, since they have different curvature, thus the content in each class and lipid species may be different, as observed. In fact multivarieted analyis showed that the lipid profile from both vesicles are different, as seen by the PCA plot. At molecular level, our results also point out a discrimination, particularly in Exo, showing the upregulation of several lipid species compared with Tropho and MVs, specially enhanced lysolipids and ceramides, well known for their relevant signaling role in modulating the inflammatory response, among others [60, 61].

Both exosomes and microvesicles contribute with their lipid molecules or their lipid-related enzymes to several pathophysiologies, including inflammation, tumor development, and atherogenesis [62].

EVs have the ability to function as carriers that can remove unwanted or toxic cellular material and transfer molecules between cells, playing various biological roles. Specifically, EVs contain bioactive lipids and enzymes responsible for their synthesis, which can affect the behaviour of recipient cells in terms of lipid transfer and metabolism [28]. Not only do EVs act as carriers for lipid mediators synthesized in releasing cells, but they can also induce the production of lipids in recipient cells, as supported by multiple studies reporting lipid-related effects mediated by EVs [63].

Lipids produced by *Schistosoma mansoni*, a helminthic parasite, were found to trigger M2 polarisation of macrophages [64] and to activate human eosinophils [65]; interestingly, some studies suggest that EVs is the most effective way for delivering these lipids [66].

In *G*. *lamblia*, lipids play important roles in its life cycle. Cholesterol, for example, plays a regulatory role in inducing encystation and its deprivation is necessary and sufficient to induce the differentiation of *Giardia* trophozoites into cysts. Evans-Osses and collaborators demonstrated the involvement of cholesterol in MV release, by observing an inhibition of MV production using different concentrations of methyl-β-cyclodextrin (MβCD). Furthermore, the absence of cholesterol inhibited the parasites’ attachment to the host cell, which was subsequently restored by the exogenous presence of MVs [7]. Also inhibitors of sphingolipids synthesis result in decreased growth and encystation of *Giardia in vitro* [67]. Sphingolipids, especially glucosylceramide, play a critical role in inducing encystation and maintaining the cyst viability in *Giardia* [68]. Sphingolipids are a highly complex class of lipids in terms of structural diversity, metabolism, and cellular functions [67]. Although once presumed to serve as inert structural constituents of eukaryotic cell membranes, sphingolipids are now recognized as playing a crucial role in signal transduction. Ceramide, a key component of sphingolipid biosynthesis, assumes an important role as bioactive lipid in signal pathways that regulates vital cellular responses such as apoptosis, cell differentiation, and cell cycle arrest in various cell types. Ceramide, LacCer and GM3 are potent signaling molecules that modify the immune response [69] and seems to contribute for the immunomodulatory effects ascribed to *Plasmodium falciparum* MVs [70]. It is also proposed that the secretion of Cer-1-P, a proinflammatory molecule, and other SL derivatives by *Giardia* could be associated with inflammatory bowel disease (IBD) observed in acute giardiasis [22]. Interestingly, there was a higher abundance of the Cer(34:1;2O), Cer (40:1;O) and Cer(41:1;2O) in *Giardia* exossomes, which may also trigger immunomodulatory effects. Indeed our results demonstrated that EVs from *G*. *lamblia* activate the innate immune response and sustains an inflamamtory response by macrophages and dendritic cells (patent PCT/IB2022/057466; ref. P1303-7WO) and Zhao and collaborators showed that *Giardia* EVs could be internalized into primary mouse peritoneal macrophages, regulate host cell innate immunity via TLR2 and NLRP3 inflammasome signaling pathways [11]. Moreover, *G*. *lamblia* membrane lipid rafts play a role in the adhesion of this organism to human enterocyte-like cells [71]. So, we could also hypothesize that the ceramide content of *Giardia* EVs can promote parasite adhesion to host cells and can also be involved in the pro-inflammatory profile of *Giardia* EVs.

Future studies focused on the composition and functional properties of *Giardia* EVs may prove crucial to understand the role of lipids in host-parasite communication. Additionally, this knowledge could shed light to identify new and specific targets in *Giardia* that could be exploited to develop novel classes of drugs to treat giardiasis.

## Supporting information

S1 Table Lipids identified by C18-LC-MS and MS/MS of total lipid extract (mass error < 5 ppm). (DOCX)

S1 Fig Phosphatidylcholine (PC) lipid species representative MS/MS spectra. (DOCX)

S2 Fig Representative MS/MS spectra of phosphatidylethanolamine (PE) lipid species. (DOCX)

S3 Fig Representative MS/MS spectra of phosphatidylglycerol (PG) lipid species. (DOCX)

S4 Fig Representative MS/MS spectra of phosphatidylinositol (PI) lipid species. (DOCX)

S5 Fig Representative MS/MS spectra of sphingomyelin (SM) lipid species. (DOCX)

S6 Fig Representation of the relative abundance (%) of each class of lipids. (DOCX)

S7 Fig Representation of the relative abundance (%) PG lipid species. (DOCX)

S8 Fig Representation of the relative abundance (%) PC lipid species. (DOCX)

S9 Fig Representation of the relative abundance (%) LPC lipid species. (DOCX)

S10 Fig Representation of the relative abundance (%) Cer lipid species. (DOCX)

S11 Fig Representation of the relative abundance (%) PE lipid species. (DOCX)

S12 Fig Representation of the relative abundance (%) LPE lipid species. (DOCX)

S13 Fig Representation of the relative abundance (%) PI lipid species. (DOCX)

S14 Fig Representation of the relative abundance (%) SM lipid species. (DOCX)
