# Supplementary material for: Lipidome of extracellular vesicles from Giardia lamblia
Source: PLoS One. 2023 Sep 8;18(9):e0291292. doi: 10.1371/journal.pone.0291292 (PMC10490865; doi:10.1371/journal.pone.0291292)
Supplement: S1 Table — (DOCX) [file pone.0291292.s001.docx]

**S1 Table. Lipids identified by C18-LC-MS and MS/MS of total lipid extract (mass error < 5 ppm)**. Observed m/z values and respective errors were checked for all samples. Lipid species are labelled as follows: AAAA (C:N) (AAAA=lipid class abbreviation; C=number of carbon atoms in fatty acid(s); N=number of double bonds). The 'O-' prefix is used for plasmanyl species to indicate the presence of an alkyl ether substituent. Underlining (‘_’) is used to describe acyl chain compositions if the sn1, sn2, sn3 and sn4 positional isomers are uncharacterized. whereas the virgule (‘/’) character is used if the acyl chain position is determined in a specific position. Asterisk (‘*’) is used to describe lipid species that could not be assigned with fatty acyl chains due to lack of spectra information.

| **Lipid species (C:N)** | **Calculated *m/z*** | **Observed *m/z*** | **Error (ppm)** | **Retention Time** | **Fatty acyl chains (C:N)** |
| --- | --- | --- | --- | --- | --- |
| **Phospholipids** | | | | | |
| **PC identified as [M+H]+** | | | | | |
| PC 30:0 | 706.5381 | 706.5394 | -1.8541 | 9.60 | 14:0_16:0 |
| PC 30:1 | 704.5225 | 704.5238 | -1.8168 | 10.63 | 14:1_16:0 |
| PC 32:0 | 734.5694 | 734.5709 | -2.0420 | 10.81 | 16:0_16:0 |
| PC 32:1 | 732.5538 | 732.5553 | -2.0886 | 9.67 | 16:0_16:1 |
| PC 32:2 | 730.5381 | 730.5397 | -2.2039 | 8.66 | * |
| PC 33:1 | 746.5694 | 746.5714 | -2.6789 | 10.28 | 16:0_17:1 |
| PC 33:2 | 744.5538 | 744.5555 | -2.3235 | 9.29 | * |
| PC 33:3 | 742.5381 | 742.5387 | -0.8215 | 8.64 | * |
| PC 34:0 | 762.6007 | 762.6025 | -2.3472 | 12.29 | 16:0_18:0 |
| PC 34:1 | 760.5851 | 760.5868 | -2.2614 | 10.87 | 16:0_18:1 |
| PC 34:2 | 758.5699 | 758.5714 | -1.8693 | 9.85 | 16:0_18:2 |
| PC 34:3 | 756.5538 | 756.5557 | -2.5510 | 9.05 | 16:0_18:3 and 16:1_18:2 |
| PC 34:4 | 754.5381 | 754.5361 | 2.6374 | 8.53 | * |
| PC 35:1 | 774.6013 | 774.6024 | -1.4433 | 11.65 | 17:0_18:1 |
| PC 35:2 | 772.5851 | 772.5867 | -2.0969 | 10.60 | 17:0_18:2 |
| PC 35:3 | 770.5694 | 770.5704 | -1.2977 | 9.84 | * |
| PC 35:4 | 768.5538 | 768.5554 | -2.1209 | 9.05 | * |
| PC 36:1 | 788.6164 | 788.6184 | -2.5488 | 12.36 | 18:0_18:1 |
| PC 36:2 | 786.6007 | 786.6027 | -2.5299 | 11.17 | 18:0_18:2 and 18:1_18:1 |
| PC 36:3 | 784.5851 | 784.5875 | -3.0844 | 9.97 | 16:0_20:3 and 18:1_18:2 |
| PC 36:4 | 782.5694 | 782.5704 | -1.2778 | 9.63 | 16:0_20:4 and 18:2_18:2 |
| PC 36:5 | 780.5543 | 780.5513 | 3.8832 | 8.83 | 16:0_20:5 |
| PC 37:3 | 798.6007 | 798.5992 | 1.8908 | 6.74 | * |
| PC 37:4 | 796.5851 | 796.5858 | -0.9039 | 10.28 | * |
| PC 38:0 | 818.6639 | 818.6624 | 1.8103 | 15.37 | 19:0_19:0 |
| PC 38:3 | 812.6164 | 812.6169 | -0.6276 | 11.53 | 18:0_20:3 |
| PC 38:4 | 810.6007 | 810.6012 | -0.6045 | 10.897 | 16:0_22:4 and 18:0_20:4 |
| PC 38:5 | 808.5851 | 808.5861 | -1.2615 | 9.68 | 16:0_22:5 |
| PC 38:6 | 806.5694 | 806.5701 | -0.8679 | 9.31 | 16:0_22:6 |
| PC 40:4 | 838.6326 | 838.6334 | -0.9754 | 11.98 | 18:0_22:4 |
| PC 40:5 | 836.6169 | 836.6171 | -0.2008 | 10.93 | 18:0_22:5 |
| PC 40:6 | 834.6007 | 834.6015 | -0.9466 | 9.86 | * |
| PC O-31:1 | 704.5589 | 704.5598 | -1.2774 | 10.79 | * |
| PC O-32:0 | 720.5902 | 720.5915 | -1.7902 | 12.04 | * |
| PC O-32:1 | 718.5745 | 718.5757 | -1.6421 | 11.670 | * |
| PC O-32:2 | 716.5589 | 716.5605 | -2.2329 | 10.42 | * |
| PC O-32:3 | 714.5432 | 714.5439 | -0.9657 | 9.29 | * |
| PC O-33:1 | 732.5902 | 732.5909 | -0.9419 | 12.20 | * |
| PC O-33:2 | 730.5745 | 730.577 | -3.3946 | 10.73 | O-15:1_18:1 |
| PC O-33:3 | 728.5589 | 728.5601 | -1.6471 | 9.82 | * |
| PC O-34:0 | 748.6215 | 748.6228 | -1.7098 | 13.71 | * |
| PC O-34:1 | 746.6057 | 746.6066 | -1.1117 | 12.09 | * |
| PC O-34:2 | 744.5902 | 744.5919 | -2.2697 | 11.69 | * |
| PC O-34:3 | 742.5745 | 742.5712 | 4.4710 | 10.575 | O-16:1_18:2 |
| PC O-35:2 | 758.6058 | 758.608 | -2.9396 | 12.23 | * |
| PC O-35:3 | 756.5902 | 756.5916 | -1.8372 | 11.03 | * |
| PC O-36:1 | 774.6371 | 774.6346 | 3.2015 | 13.81 | O-18:0_18:1 |
| PC O-36:2 | 772.6215 | 772.6235 | -2.5627 | 12.27 | * |
| PC O-36:3 | 770.6058 | 770.6068 | -1.3366 | 11.90 | * |
| **LPC identified as [M+H]+** | | | | | |
| LPC 15:0 | 482.3247 | 482.3249 | -0.4831 | 2.45 | 15:0 |
| LPC 16:0 | 496.3403 | 496.3408 | -0.9751 | 2.93 | 16:0 |
| LPC 16:1 | 494.3247 | 494.3252 | -1.0782 | 2.29 | 16:1 |
| LPC 17:0 | 510.3554 | 510.3568 | -2.7236 | 3.35 | 17:0 |
| LPC 18:0 | 524.3716 | 524.373 | -2.6374 | 3.96 | 18:0 |
| LPC 18:1 | 522.3559 | 522.3569 | -1.7861 | 3.09 | 18:1 |
| LPC 18:2 | 520.3403 | 520.3411 | -1.5067 | 2.45 | 18:2 |
| LPC 20:0 | 552.4029 | 552.4041 | -2.1415 | 4.11 | 20:0 |
| LPC 20:4 | 544.3403 | 544.3411 | -1.4403 | 2.34 | 20:4 |
| LPC O-16:1 | 480.3454 | 480.3464 | -2.0777 | 3.41 | O-16:1 |
| **PE identified as [M-H]-** | | | | | |
| PE 31:0 | 676.4917 | 676.4940 | -3.3526 | 10.32 | 15:0_16:0 |
| PE 31:1 | 674.4761 | 674.4781 | -2.9919 | 9.02 | * |
| PE 32:0 | 690.5074 | 690.5092 | -2.6328 | 10.96 | 16:0_16:0 |
| PE 32:1 | 688.4917 | 688.4933 | -2.2774 | 9.82 | 16:0_16:1 |
| PE 32:2 | 686.4761 | 686.4784 | -3.3767 | 8.77 | 16:1_16:1 |
| PE 33:0 | 704.5230 | 704.5244 | -1.9417 | 11.65 | 15:0_18:0 and 16:0_17:0 |
| PE 33:1 | 702.5074 | 702.5099 | -3.5843 | 10.40 | 15:0_18:1 |
| PE 33:2 | 700.4917 | 700.4937 | -2.8094 | 9.43 | 15:0_18:2 |
| PE 34:0 | 718.5387 | 718.5400 | -1.8357 | 12.34 | 16:0_18:0 |
| PE 34:1 | 716.5230 | 716.5237 | -0.9323 | 11.04 | 16:0_18:1 |
| PE 34:2 | 714.5074 | 714.5074 | -0.0252 | 9.86 | 16:0_18:2 and 16:1_18:1 |
| PE 34:3 | 712.4917 | 712.4943 | -3.6042 | 9.20 | 16:0_18:3 |
| PE 35:1 | 730.5392 | 730.5405 | -1.8069 | 11.95 | 16:0_19:1 and 17:0_18:1 |
| PE 35:2 | 728.52362 | 728.5244 | -1.0707 | 10.66 | 17:1_18:1 |
| PE 36:1 | 744.55433 | 744.5558 | -1.9716 | 12.40 | 18:0_18:1 |
| PE 36:2 | 742.5387 | 742.5397 | -1.3723 | 11.09 | 18:1_18:1 SN1/SN2 |
| PE 36:3 | 740.5230 | 740.5242 | -1.5773 | 10.18 | 18:1_18:2 |
| PE O-34:2 | 742.5751 | 742.5748 | 0.3596 | 11.13 | * |
| PE O-37:2 | 700.5287 | 700.5314 | -3.8685 | 11.81 | O-16:1_18:1 |
| **LPE identified as [M-H]-** | | | | | |
| LPE 18:0 | 480.3096 | 480.3103 | -1.4574 | 3.99 | 18:0 |
| LPE 18:1 | 478.2939 | 478.2945 | -1.2335 | 3.00 | 18:1 |
| **PG identified as [M-H]-** | | | | | |
| PG 30:0 | 693.4710 | 693.4727 | -2.8840 | 8.49 | 14:0_16:0 |
| PG 31:0 | 707.4863 | 707.4888 | -3.5152 | 9.07 | 15:0_16:0 and 14:0_17:0 |
| PG 32:0 | 721.5020 | 721.5030 | -1.3860 | 9.64 | 16:0_16:0 |
| PG 32:1 | 719.4863 | 719.4857 | 0.8339 | 8.61 | 16:0_16:1 |
| PG 33:0 | 735.5176 | 735.5187 | -1.4779 | 10.23 | 16:0_17:0 and 15:0_18:0 |
| PG 33:1 | 733.5020 | 733.5025 | -0.7335 | 9.16 | 15:0_18:1 and 16:0_17:0 |
| PG 34:0 | 749.5333 | 749.5337 | -0.5337 | 10.82 | 16:0_18:0 SN1/SN2 |
| PG 34:1 | 747.5176 | 747.5185 | -1.2040 | 9.73 | 16:0_18:1 |
| PG 34:2 | 745.5020 | 745.5036 | -2.1462 | 8.85 | 16:1_18:1 SN1/SN2 |
| PG 35:0 | 763.5489 | 763.5505 | -2.0784 | 11.22 | 17:0_18:0 |
| PG 35:2 | 759.5176 | 759.5161 | 1.9921 | 9.43 | 17:1_18:1 |
| PG 36:0 | 777.5650 | 777.5668 | -2.8293 | 12.10 | 16:0_20:0 |
| PG 36:1 | 775.5490 | 775.5479 | 1.2894 | 10.91 | 18:0_18:1 |
| PG 36:2 | 773.5330 | 773.5341 | -1.0342 | 9.78 | 18:1_18:1 |
| PG 36:3 | 771.5180 | 771.5195 | -2.4627 | 8.93 | 18:1_18:2 |
| PG 36:4 | 769.5020 | 769.5040 | -2.5991 | 8.09 | 18:2_18:2 |
| **LPG identified as [M-H]-** | | | | | |
| LPG 14:0 | 455.2410 | 455.2424 | -3.0797 | 1.87 | 14:0 |
| **PI identified as [M-H]-** | | | | | |
| PI 32:1 | 807.5029 | 807.5024 | 0.6563 | 8.24 | 16:0_16:1 |
| PI 34:0 | 837.5499 | 837.5513 | -1.6357 | 10.39 | 16:0_18:0 |
| PI 34:1 | 835.5342 | 835.5350 | -0.9814 | 9.34 | 16:0_18:1 |
| PI 34:2 | 833.5186 | 833.5207 | -2.4954 | 8.40 | 16:1_18:1 |
| PI 36:1 | 863.5655 | 863.5672 | -1.9802 | 10.50 | 18:0_18:1 |
| **CL identified as [M-H]-** | | | | | |
| CL 66:2 | 1375.9650 | 1375.9631 | 1.3809 | 17.30 | * |
| CL 70:4 | 1427.9963 | 1427.9961 | 0.1401 | 17.20 | * |
| CL 72:4 | 1456.0276 | 1456.0232 | 3.0219 | 17.62 | * |
| **Sphingolipids** | | | | | |
| **Cer identified as [M+H]+** | | | | | |
| Cer 26:1;3O | 442.3891 | 442.3900 | -2.0344 | 7.58 | 12:0;2O/14:1;O |
| Cer 28:1;3O | 470.4204 | 470.4216 | -2.5296 | 7.18 | 14:0;2O/14:1;O |
| Cer 32:0;2O | 512.5037 | 512.5048 | -2.1073 | 10.36 | 16:0;2O/16:0 |
| Cer 32:1;2O | 510.4881 | 510.4886 | -0.9795 | 9.86 | 16:1;2O/16:0 |
| Cer 32:1;3O | 526.4830 | 526.4843 | -2.5262 | 9.77 | 14:0;2O/18:1;O |
| Cer 34:0;2O | 540.5350 | 540.5363 | -2.4605 | 11.78 | 18:0;2O/16:0 |
| Cer 34:1;2O | 538.5194 | 538.5203 | -1.6527 | 11.21 | 18:1;2O/16:0 |
| Cer 34:2;2O | 536.5037 | 536.5048 | -2.0130 | 11.34 | 19:0;2O/15:2 |
| Cer 36:1;2O | 566.5507 | 566.5519 | -2.0828 | 12.69 | 18:1;2O/18:0 |
| Cer 36:2;2O | 564.5350 | 564.5364 | -2.5331 | 11.42 | 18:2;2O/18:0 |
| Cer 37:1;2O | 580.5663 | 580.5677 | -2.4459 | 13.41 | 18:1;2O/19:0 |
| Cer 38:1;2O | 594.5820 | 594.5834 | -2.4050 | 14.05 | 18:1;2O/20:0 |
| Cer 39:1;2O | 608.5976 | 608.5992 | -2.6290 | 14.55 | 16:1;2O/23:0 |
| Cer 40:1;2O | 622.6133 | 622.615 | -2.7625 | 14.99 | 18:1;2O/22:0 |
| Cer 40:2;2O | 620.5976 | 620.5965 | 1.7725 | 13.97 | 16:1;2O/24:1 |
| Cer 41:0;2O | 638.6446 | 638.6465 | -2.9907 | 15.66 | 18:0;2O/23:0 |
| Cer 41:0;3O | 654.6395 | 654.6407 | -1.7872 | 14.99 | 17:0;2O/24:0;O |
| Cer 41:1;2O | 636.6289 | 636.6309 | -3.1258 | 15.36 | 19:1;2O/22:0 |
| Cer 42:0;2O | 652.6602 | 652.662 | -2.7273 | 15.98 | 18:0;2O/24:0 |
| Cer 42:0;4O | 684.6501 | 684.6503 | -0.3067 | 15.02 | 18:0;3O/24:0;2OH |
| Cer 42:1;2O | 650.6446 | 650.6465 | -2.9355 | 15.71 | 19:1;2O/23:0 |
| Cer 42:2;2O | 648.6289 | 648.6308 | -2.9138 | 14.92 | 18:2;2O/24:0 |
| Cer 42:3;2O | 646.6133 | 646.6152 | -2.9693 | 14.10 | 18:1;2O/24:2 |
| Cer 43:0;2O | 666.6759 | 666.6781 | -3.2999 | 16.28 | 19:0;2O/24:0 |
| Cer 43:0;4O | 698.6657 | 698.6669 | -1.7032 | 15.39 | 18:0;3O/25:0;2OH |
| Cer 43:1;2O | 664.6602 | 664.6628 | -3.8817 | 16.03 | 19:1;2O/24:0 |
| Cer 44:0;4O | 712.6814 | 712.6824 | -1.4031 | 15.72 | 18:0;3O/26:0;2OH |
| Cer 44:1;2O | 678.6759 | 678.679 | -4.5677 | 16.33 | 20:1;2O/24:0 |
| Cer 44:2;3O | 692.6551 | 692.6563 | -1.7469 | 14.85 | 19:2;2O/25:0;O |
| Cer 45:0;2O | 694.7072 | 694.7083 | -1.5690 | 16.83 | 20:0;2O/25:0 |
| Cer 45:0;3O | 710.7021 | 710.7026 | -0.7176 | 16.32 | 19:0;2O/26:0;O |
| Cer 45:1;2O | 692.6915 | 692.6911 | 0.6208 | 16.59 | 20:1;2O/25:0 |
| Cer 46:0;4O | 740.7127 | 740.7139 | -1.6066 | 16.31 | 20:0;3O/26:0;2OH |
| Cer 46:1;2O | 706.7072 | 706.7101 | -4.0894 | 16.86 | 20:1;2O/26:0 |
| HexCer 34:1;2O | 700.5722 | 700.5746 | -3.4258 | 9.79 | 18:1;2O/16:0 |
| HexCer 40:1;2O | 784.6661 | 784.6674 | -1.6822 | 13.79 | 18:1;2O/22:0 |
| HexCer 42:1;2O | 812.6974 | 812.6982 | -0.9967 | 14.76 | 18:1;2O/24:0 |
| **SM identified as [M+H]+** | | | | | |
| SM 32:1;2O | 675.5436 | 675.5464 | -4.1744 | 8.15 | 16:1;2O/16:0 |
| SM 33:1;2O | 689.5592 | 689.5599 | -1.0151 | 8.83 | 17:1;2O/16:0 |
| SM 34:0;2O | 705.5905 | 705.5917 | -1.6724 | 9.92 | * |
| SM 34:1;2O | 703.5749 | 703.5765 | -2.2883 | 9.42 | 18:1;2O/16:0 |
| SM 34:2;2O | 701.5598 | 701.5582 | 2.2108 | 8.35 | * |
| SM 36:1;2O | 731.6062 | 731.608 | -2.4603 | 10.81 | 18:1;2O/18:0 |
| SM 38:0;O2 | 761.6531 | 761.6545 | -1.8644 | 12.91 | * |
| SM 38:1;2O | 759.6375 | 759.6398 | -3.0146 | 12.24 | * |
| SM 38:2;2O | 757.6218 | 757.6251 | -4.3161 | 10.94 | * |
| SM 38:5;3O | 767.5698 | 767.5667 | 4.0648 | 10.36 | * |
| SM 40:1;2O | 787.6688 | 787.6699 | -1.3711 | 13.79 | * |
| SM 40:2;2O | 785.6531 | 785.655 | -2.4438 | 12.39 | * |
| SM 41:1;2O | 801.6844 | 801.6861 | -2.1330 | 14.35 | 18:1;2O/23:0 |
| SM 41:2;2O | 799.6688 | 799.6707 | -2.3510 | 13.27 | * |
| SM 42:1;2O | 815.7007 | 815.7014 | -1.6305 | 14.84 | * |
| SM 42:2;2O | 813.6844 | 813.6862 | -2.2244 | 13.65 | * |
| SM 42:3;2O | 811.6688 | 811.6696 | -0.9610 | 12.34 | * |
| SM 43:1;2O | 829.7157 | 829.7176 | -2.2899 | 15.33 | * |
| SM 43:2;2O | 827.7001 | 827.7018 | -2.0901 | 14.16 | * |
| **Sterol** | | | | | |
| **ST identified as [M+H-H_2_O]+** | | | | | |
| ST 27:1;O | 369.3515 | 369.3521 | -1.3808 | 18.95 | * |
