# Supplementary material for: Lipidome of extracellular vesicles from Giardia lamblia
Source: PLoS One. 2023 Sep 8;18(9):e0291292. doi: 10.1371/journal.pone.0291292 (PMC10490865; doi:10.1371/journal.pone.0291292)
Supplement: S1 Fig — (DOCX) [file pone.0291292.s002.docx]

**S1 Fig.** **Phosphatidylcholine (PC) lipid species representative MS/MS spectra.**


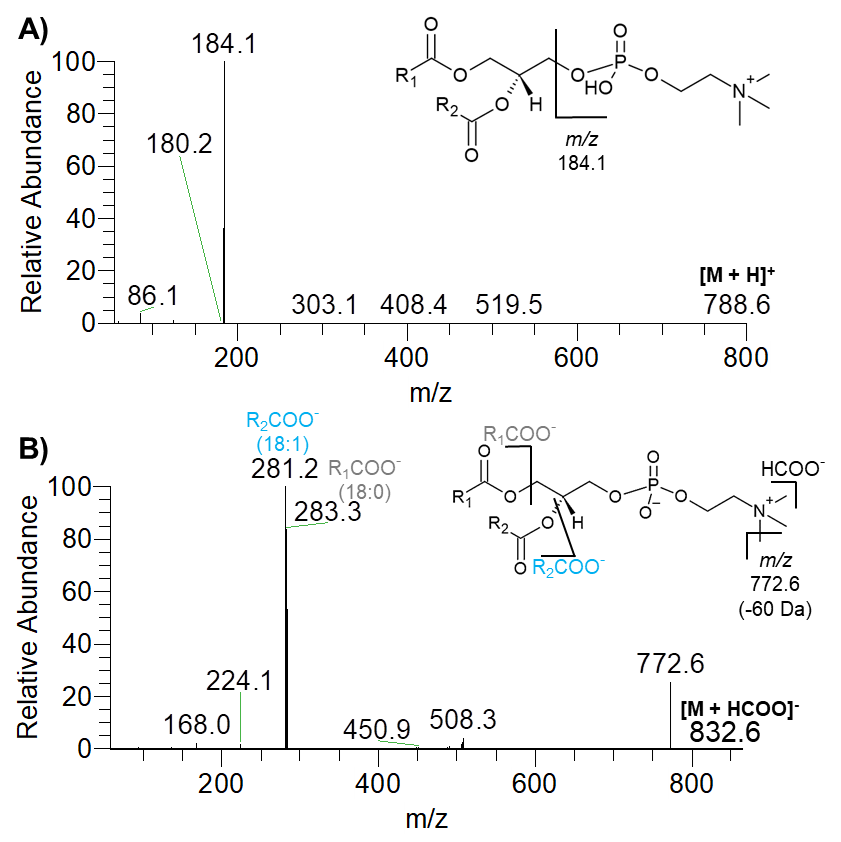


Supplementary Figure S2. Phosphatidylcholine (PC) lipid species representative MS/MS spectra**.** A) The C18-LC-MS/MS spectrum of the lipid specie PC 32:1 observed in positive mode as [M + H]^+^ ion at m/z 788.6. Confirmation of phospholipid class was achieved by the identification of the product ion at m/z 184.1 (formula: C_5_H_15_NO_4_P; exact mass: 184.0739), corresponding to the phosphocholine polar head. **B)** The C18-LC-MS/MS spectrum of the lipid specie PC 32:1 observed in negative mode as [M + HCOO]^-^ ion at m/z 832.6. Confirmation of phospholipid class was achieved by observing the characteristic neutral loss of 60 Da (formula: C_2_H_4_O_2_; exact mass: 60.0211), corresponding to the loss of methyl acetate. Fatty acid composition was confirmed by the identification of product ions corresponding to the fatty acyl chains as [RCOO]^-^. In this case, two molecular lipid species with distinct fatty acyl composition were identified. The product ions observed at m/z 283.3 and 281.2, corresponding to fatty acyl carboxylate anions 18:0 (R_1_COO^-^) and 18:1 (R_2_COO^-^) allowed to identify the fatty acyl composition of PC 18:0_18:1. For LPC, the same fragmentation was observed, with the exception that only one product ion corresponding to a fatty acid was detected
