# Supplementary material for: Lipidome of extracellular vesicles from Giardia lamblia
Source: PLoS One. 2023 Sep 8;18(9):e0291292. doi: 10.1371/journal.pone.0291292 (PMC10490865; doi:10.1371/journal.pone.0291292)
Supplement: S2 Fig — (DOCX) [file pone.0291292.s003.docx]

**S2 Fig. Representative MS/MS spectra of phosphatidylethanolamine (PE) lipid species.**


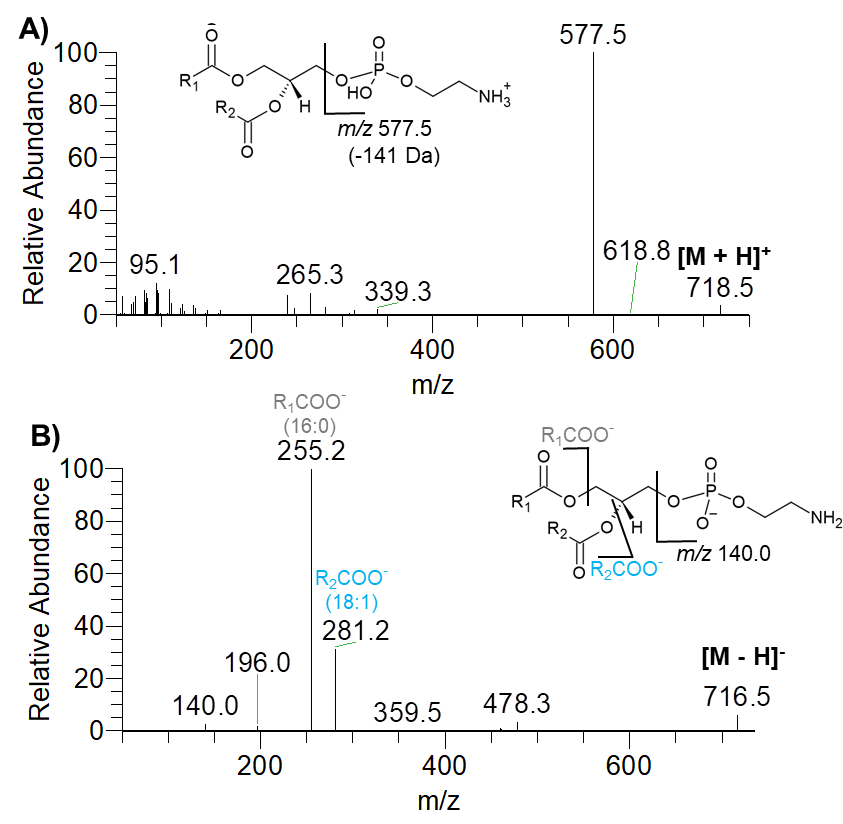


**Supplementary Figure S2.** Representative MS/MS spectra of phosphatidylethanolamine (PE) lipid species. **A)** The C18-LC-MS/MS spectrum of the PE 34:1 observed in positive mode as [M + H]^+^ ion at m/z 718.5. Confirmation of phospholipid class was achieved by the identification of the neutral loss of 141 Da (formula: C2H8NO4P; exact mass: 141.0191), corresponding to phosphoethanolamine polar head. **B)** The C18-LC-MS/MS spectrum of the lipid specie 34:1 observed in negative mode as [M - H]^-^ at m/z 716.5. Fatty acid composition was confirmed by the identification of product ions corresponding to the fatty acyl chains as [RCOO]^-^. The product ions observed at m/z 255.2 and 281.2.2, corresponding to fatty acyl carboxylate anions of 16:0 (R_1_COO^-^) and 18.1 (R_2_COO^-^), allowed to identify the fatty acyl composition of PE 16:0_18:1. The confirmation as PE species was also achieved by observing the product ion at m/z 140.0 (formula: C2H7NO4P; exact mass: 140.0113), corresponding to phosphoethanolamine polar head. For LPE, the same fragmentation was observed, with the exception that only one product ion corresponding to a fatty acid was detected
