# Supplementary material for: Lipidome of extracellular vesicles from Giardia lamblia
Source: PLoS One. 2023 Sep 8;18(9):e0291292. doi: 10.1371/journal.pone.0291292 (PMC10490865; doi:10.1371/journal.pone.0291292)
Supplement: S3 Fig — (DOCX) [file pone.0291292.s004.docx]

**S3 Fig. Representative MS/MS spectra of phosphatidylglycerol (PG) lipid species.**


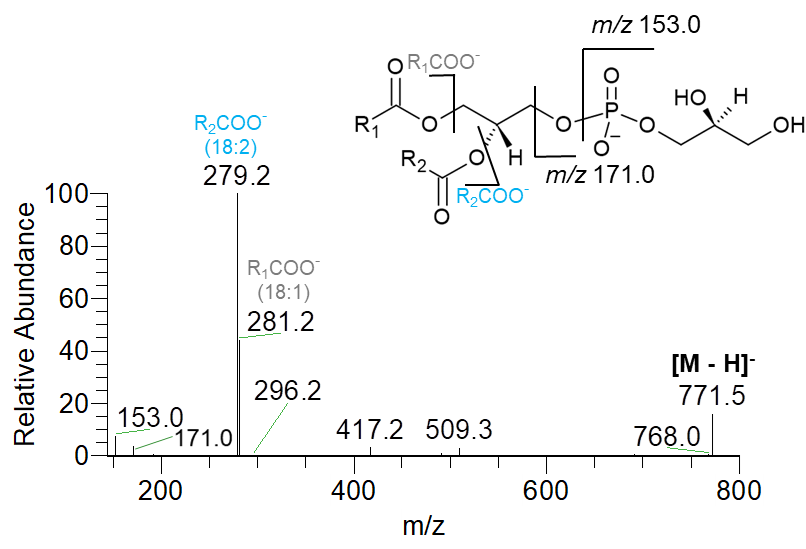


Supplementary Figure S3. Representative MS/MS spectra of phosphatidylglycerol (PG) lipid species. The C18-LC-MS/MS spectrum of the PG 36:3 observed in negative mode as [M - H]^-^ at m/z 771.5. Confirmation of phospholipid class may be achieved by the identification of the product ion at m/z 171.0 (formula: C3H8O6P; exact mass: 171.0058), corresponding to the glycerol phosphate anion. Fatty acid composition was confirmed by the identification of product ions corresponding to the fatty acyl chains as [RCOO]^-^. The product ions observed at m/z 281.2 and 279.2, corresponding to fatty acyl carboxylate anions of 18:1 (R_1_COO^-^) and 18.2 (R_2_COO-), allowed to identify the fatty acyl composition of PG 18:1_18:2. For LPG, the same fragmentation was observed, with the exception that only one product ion corresponding to a fatty acid was detected.
