# Supplementary material for: Lipidome of extracellular vesicles from Giardia lamblia
Source: PLoS One. 2023 Sep 8;18(9):e0291292. doi: 10.1371/journal.pone.0291292 (PMC10490865; doi:10.1371/journal.pone.0291292)
Supplement: S4 Fig — (DOCX) [file pone.0291292.s005.docx]

**S4 Fig. Representative MS/MS spectra of phosphatidylinositol (PI) lipid species.**


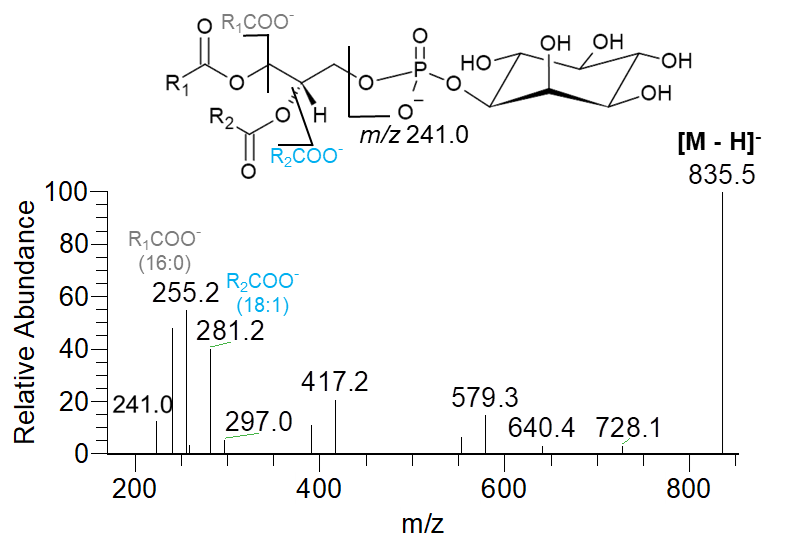


Supplementary Figure S4. Representative MS/MS spectra of phosphatidylinositol (PI) lipid species. The C18-LC-MS/MS spectrum of the PI 34:1 observed in negative mode as [M - H]^-^ at m/z 835.5. Confirmation of phospholipid class may be achieved by the identification of the product ion at m/z 241.0 (formula: C6H10O8P; exact mass: 241.0113), corresponding to the phosphoinositol head group. Fatty acid composition was confirmed by the identification of product ions corresponding to the fatty acyl chains as [RCOO]^-^. The product ions observed at m/z 255.2 and 281.2, corresponding to fatty acyl carboxylate anions of 16:0 (R_1_COO^-^) and 18:1 (R_2_COO^-^), allowed to identify the fatty acyl composition of PI 16:0_18:1.
