# Supplementary material for: Lipidome of extracellular vesicles from Giardia lamblia
Source: PLoS One. 2023 Sep 8;18(9):e0291292. doi: 10.1371/journal.pone.0291292 (PMC10490865; doi:10.1371/journal.pone.0291292)
Supplement: S5 Fig — (DOCX) [file pone.0291292.s006.docx]

**S5 Fig. Representative MS/MS spectra of sphingomyelin (SM) lipid species.**


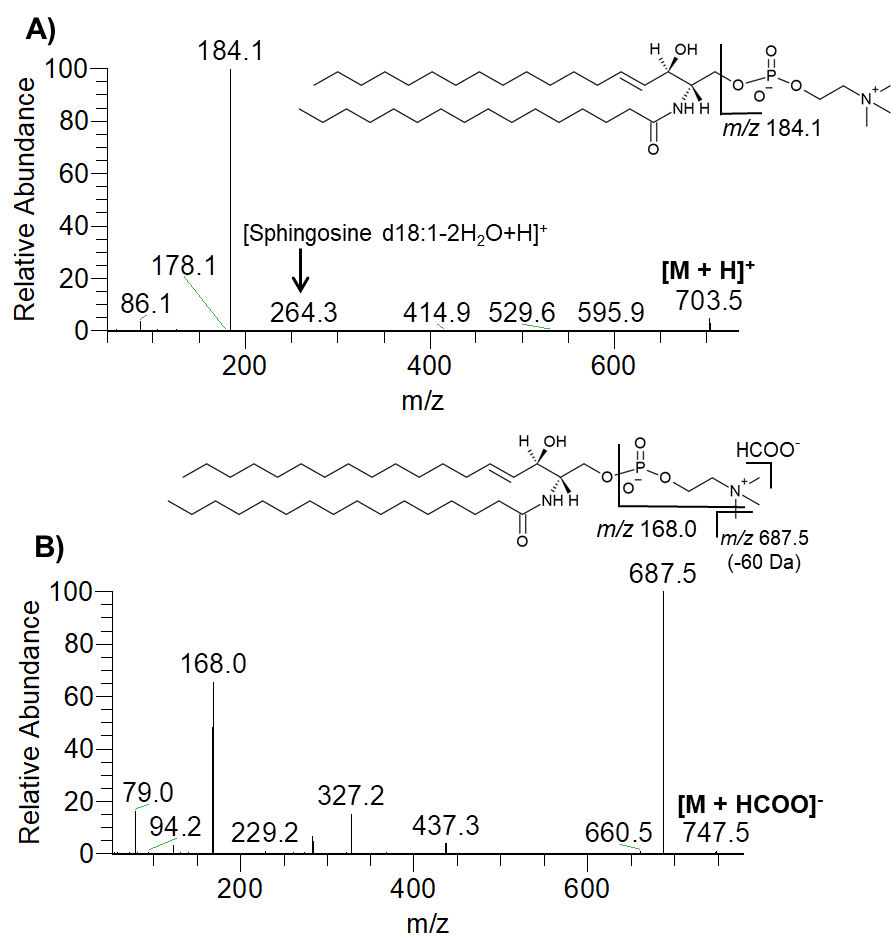


Supplementary Figure S5. Representative MS/MS spectra of sphingomyelin (SM) lipid species. **A)** The C18-LC-MS/MS spectrum of the SM d34:1 observed in positive mode as [M + H]^+^ ion at m/z 703.5. Confirmation of sphingolipid class was achieved by the identification of the product ion at m/z 184.1 (formula: C5H15NO4P; exact mass: 184.0739), corresponding to the phosphocholine polar head; and the product ion of sphingoid base at 264.3, corresponding to [Sphingosine d18:1-2H2O+H]^+^. **B)** The C18-LC-MS/MS spectrum of the lipid specie SM d34:1 observed in negative mode as [M + HCOO]^-^ ion at m/z 747.5. Confirmation of phospholipid class was achieved by the identification of the product ion at m/z 168.0 (formula: C4H11NO4P; exact mass:168.0425), corresponding to the phosphocholine polar head without a methyl group, and by observing the characteristic neutral loss of 60 Da (formula: C2H4O2; exact mass: 60.021130), corresponding to the loss of methyl formate.
