# Supplementary material for: Lipidome of extracellular vesicles from Giardia lamblia
Source: PLoS One. 2023 Sep 8;18(9):e0291292. doi: 10.1371/journal.pone.0291292 (PMC10490865; doi:10.1371/journal.pone.0291292)
Supplement: S6 Fig — (DOCX) [file pone.0291292.s007.docx]

**S6 Fig. Representation of the relative abundance (%) of each class of lipids.**


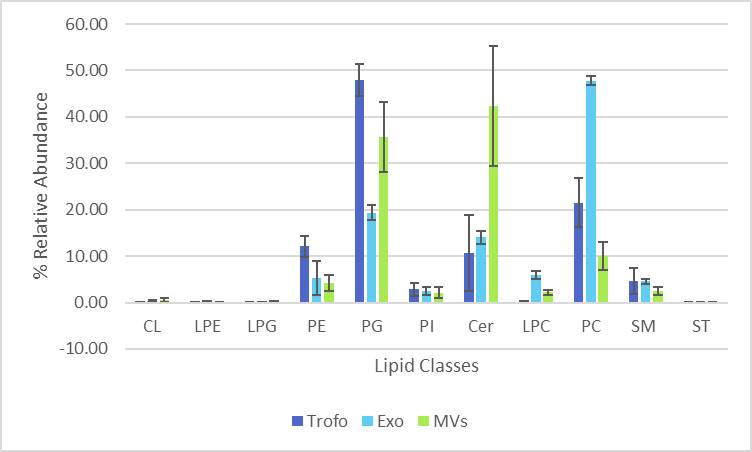


Supplementary Figure S6. Representation of the relative abundance (%) of each class of lipids (calculated by dividing each lipid class (µg) (calculated by the sum of all lipid species per class) per sum of all lipid classes (µg)). The highest levels were observed for PG, PC and Cer classes in all of three groups (Trofo, MVs and Exo).
