# Supplementary material for: Lipidome of extracellular vesicles from Giardia lamblia
Source: PLoS One. 2023 Sep 8;18(9):e0291292. doi: 10.1371/journal.pone.0291292 (PMC10490865; doi:10.1371/journal.pone.0291292)
Supplement: S7 Fig — (DOCX) [file pone.0291292.s008.docx]

**S7 Fig. Representation of the relative abundance (%) PG lipid species**.


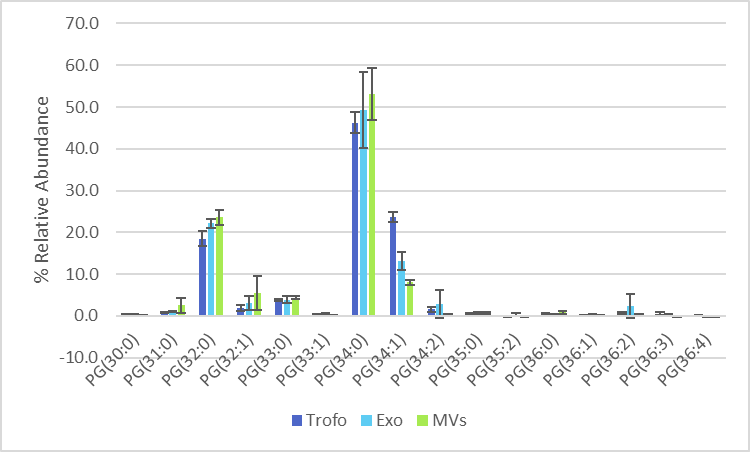


Supplementary Figure S7. Representation of the relative abundance (%) PG lipid species (calculated by dividing each lipid species (µg) by the sum of all lipid species of the same class (µg))
